# Combined Fluorescence and Optoacoustic Imaging for Monitoring Treatments against CT26 Tumors with Photoactivatable Liposomes

**DOI:** 10.3390/cancers14010197

**Published:** 2021-12-31

**Authors:** Ilya Turchin, Shazia Bano, Mikhail Kirillin, Anna Orlova, Valeriya Perekatova, Vladimir Plekhanov, Ekaterina Sergeeva, Daria Kurakina, Aleksandr Khilov, Alexey Kurnikov, Pavel Subochev, Marina Shirmanova, Anastasiya Komarova, Diana Yuzhakova, Alena Gavrina, Srivalleesha Mallidi, Tayyaba Hasan

**Affiliations:** 1 Institute of Applied Physics RAS, 46 Ulyanov St., 603950 Nizhny Novgorod, Russia; mkirillin@yandex.ru (M.K.); ag.orlova@mail.ru (A.O.); Valeriya1000@yandex.ru (V.P.); plehanov_vi@mail.ru (V.P.); sea@ufp.appl.sci-nnov.ru (E.S.); vekfy@inbox.ru (D.K.); alhil@inbox.ru (A.K.); kurnikov.1997@mail.ru (A.K.); pavel.subochev@gmail.com (P.S.); 2 Wellman Center for Photomedicine, Massachusetts General Hospital and Harvard Medical School, Boston, MA 02114, USA; sbano@mgh.harvard.edu (S.B.); Srivalleesha.mallidi@tufts.edu (S.M.); thasan@mgh.harvard.edu (T.H.); 3 Institute of Experimental Oncology and Biomedical Technologies, Privolzhsky Research Medical University, 10/1 Minin and Pozharsky Sq., 603005 Nizhny Novgorod, Russia; shirmanovam@mail.ru (M.S.); leeemonk76g@gmail.com (A.K.); yuzhakova-diana@mail.ru (D.Y.); gavrina.alena@mail.ru (A.G.); 4 Department of Biomedical Engineering, Tufts University, Medford, MA 02155, USA; 5 Division of Health Sciences and Technology, Harvard University and Massachusetts Institute of Technology, Cambridge, MA 02139, USA

**Keywords:** optoacoustic imaging, fluorescence imaging, photodynamic therapy, multi-inhibitor liposomes, tumor vasculature, nanoconstructs

## Abstract

**Simple Summary:**

A new approach of combined optoacoustic (OA) and fluorescence (FL) imaging for in vivo real-time tracing photosensitizer (PS) kinetics and functional vascular effects in the treated area was developed. FL monitoring using a photoactivatable multi-inhibitor liposomal (PMILs) platform, demonstrates enhancement of PS accumulation in tumor, 24 h post-treatment. OA monitoring revealed the alterations of the tumor vasculature structure after treatment, which is in good agreement with the histological data that shows five times higher percentage of hemorrhages in PMIL treated mice compared to the untreated group.

**Abstract:**

The newly developed multimodal imaging system combining raster-scan optoacoustic (OA) microscopy and fluorescence (FL) wide-field imaging was used for characterizing the tumor vascular structure with 38/50 μm axial/transverse resolution and assessment of photosensitizer fluorescence kinetics during treatment with novel theranostic agents. A multifunctional photoactivatable multi-inhibitor liposomal (PMILs) nano platform was engineered here, containing a clinically approved photosensitizer, Benzoporphyrin derivative (BPD) in the bilayer, and topoisomerase I inhibitor, Irinotecan (IRI) in its inner core, for a synergetic therapeutic impact. The optimized PMIL was anionic, with the hydrodynamic diameter of 131.6 ± 2.1 nm and polydispersity index (PDI) of 0.05 ± 0.01, and the zeta potential between −14.9 ± 1.04 to −16.9 ± 0.92 mV. In the in vivo studies on BALB/c mice with CT26 tumors were performed to evaluate PMILs’ therapeutic efficacy. PMILs demonstrated the best inhibitory effect of 97% on tumor growth compared to the treatment with BPD-PC containing liposomes (PALs), 81%, or IRI containing liposomes (L-[IRI]) alone, 50%. This confirms the release of IRI within the tumor cells upon PMILs triggering by NIR light, which is additionally illustrated by FL monitoring demonstrating enhancement of drug accumulation in tumor initiated by PDT in 24 h after the treatment. OA monitoring revealed the largest alterations of the tumor vascular structure in the PMILs treated mice as compared to BPD-PC or IRI treated mice. The results were further corroborated with histological data that also showed a 5-fold higher percentage of hemorrhages in PMIL treated mice compared to the control groups. Overall, these results suggest that multifunctional PMILs simultaneously delivering PDT and chemotherapy agents along with OA and FL multi-modal imaging offers an efficient and personalized image-guided platform to improve cancer treatment outcomes.

## 1. Introduction

Imaging plays a major role in the development of effective cancer therapies. Specifically, monitoring drug delivery, and dynamic changes in physical parameters, such as blood flow, tumor oxygenation, and vessel structure, is key for evaluating therapeutic efficacy. Amongst several therapies being developed, Photodynamic therapy (PDT) is a therapeutic approach that is gaining wide recognition in clinical practice. It is based on irradiation of photosensitizers (PS) with the light of a certain wavelength in the presence of oxygen in a tissue resulting in the formation of cytotoxic reactive oxygen species (ROS) [1,2,3]. In addition to the direct injury of cancer cells by the PDT, the destruction of the tumor microcirculatory system, mediating the killing of tumor cells indirectly, is also considered as one of the main mechanisms of PDT [2,4]. Photodynamic destruction of endothelial cells interrupts local microcirculation and prevents the supply of oxygen and nutrients to the tumor. As a result, functional reactions of tumor vessels, such as vasoconstriction, occlusions, thrombosis, and hypoxia develop [5]. Alterations to blood flow after the so-called “vascular” PDT are considered as the criteria of tumor response to treatment [6,7,8,9,10]; therefore, monitoring of these parameters can be important for the development of novel PDT regimens. Vascular PDT has been shown to be efficient for cancer treatment [11,12,13,14,15]. It can be implemented by a short drug-to-light interval when the irradiation is carried out at the time when the photosensitizer is predominantly located in the blood and has not yet been or minimally distributed to the surrounding tissues. Understanding the importance of treatment-induced tumor vascular changes attracted the non-invasive methods for monitoring the structural and functional characteristics of the microcirculatory system after PDT alone or in combination with other therapies [10,16,17,18,19,20].

Different techniques for monitoring blood flow perfusion, tumor oxygenation, and vessel structure were reported for monitoring the effect of PDT on the tumor microcirculatory system. Blood flow perfusion has been investigated with different modalities [3,21,22,23]: Laser Doppler and Laser Speckle Imaging, Dynamic Optical Coherence Tomography, and Doppler Ultrasound. Tissue oxygen state is analyzed in vivo using Electron Paramagnetic Resonance Imaging, Diffuse Optical Spectroscopy, Optoacoustic (OA) imaging [7,15,24,25,26]. Structural imaging can be performed using Magnetic Resonance Imaging, CT angiography, and OA imaging, which has rapidly emerged in the last decade [27]. OA imaging is a hybrid technique with the advantages of optics and ultrasonics [28,29]. One of the main applications of OA imaging in experimental oncology is the analysis of tumor vascular patterns during natural growth [27,30], as well as under various therapies [24,31,32] or functional tests [33,34,35]. OA has been recently used for monitoring changes in vascular structure and blood oxygenation following PDT [23]. Tumor response to HPPH-based PDT included a time-dependent decrease in vessel diameter and vascular area [36]; such vascular shutdown was also revealed by OA after PDT with ICG-lactosome [37]. Rapid vessel occlusion and hemorrhage formation were demonstrated in response to treatment with Padeliporfin [38].

The ability of most PS to emit rather high fluorescence under light excitation at the specific wavelength made the PDT combined with fluorescence (FL) imaging a real theranostic approach [10,39,40,41,42]. This relatively simple and inexpensive technique of FL imaging is widely used in preclinical and clinical studies to monitor PDT [43]. It allows to control PS accumulation in tumor [42,44,45,46] and surrounding tissues after drug administration and tracks PS photobleaching during laser irradiation [39] and PS circulation after the procedure [47]. Most of the modern studies aimed at improving the PDT efficiency are directed at the development of novel photosensitizers [48,49], validation of new protocols of tumor irradiation [10], a combination of PDT with other methods of anticancer treatment [50,51,52,53], and development of new approaches for monitoring post-PDT tumor responses [9,10,54]. Meanwhile, there is a lack of reliable, non-invasive techniques to monitor the therapeutic processes and tumor response in vivo simultaneously.

Colorectal cancer (CRC) ranks third for incidence worldwide, with a median overall survival of six to eight months. Currently, FOLFIRI (FOL: Leucovorin Calcium, F: Fluorouracil, IRI: Irinotecan) is an approved regimen used in clinical practice as a major treatment for CRC [55]. In spite of chemotherapy efficacy, its use is hindered by several unpleasant dose-limiting side effects. The high rate of toxicity has promoted the development of transformative approaches to better manage the disease [56,57]. As such, the combination of chemotherapeutic drugs may be improved by formulating liposome-encapsulated versions that reduce toxicity and benefit survival, such as the successful application of liposomal IRI in metastatic pancreatic cancer treatments. Due to the inherent ability to carry high payloads within a single entity, preferential accumulation of liposomes results in the localization of a greater amount of the theranostic within the tumor [50], integrating diagnosis with the treatment process. The significant contribution of nanoliposome-based drug delivery systems is known for many clinical products, including Doxil^®^, Ambisome^®^, DepoDur™, and ONIVYDE^®^, etc. [58]. Onivyde (a nanoliposomal formulation of IRI) has shown improved pharmacokinetics and biodistribution of the drug by increasing drug loading efficiency, protecting the drug from premature metabolism, extending circulation time, providing sustained release, and minimizing the host toxicity [59,60]. First-line NALIRIFOX (liposomal IRI) raised no new safety signals in patients with locally advanced/metastatic pancreatic cancer. However, the phase III NAPOLI-3 study (NCT04083235) will compare NALIRIFOX in combination with gemcitabine and paclitaxel [61].

It has also been reported that PDT treatment with PS and chemotherapeutic drugs loaded with liposomes induced tumor growth inhibition more effectively [62,63,64]. The combination of PS with other drugs helps to overcome multidrug resistance, destroys tumor cells that have survived after PDT action, and prevents revascularization [62,65]. As a fact, when a combination of photosensitizers and chemotherapeutic drugs is used, PDT induces direct tumor cell death and sensitization to chemotherapy. Thus PDT-induced blood flow disturbance can contribute to the accumulation of the chemotherapeutic agents in the treated area [66]. Our recently published work has established a tunable, modular nanochemistry approach to deliver photodynamic therapy, to the heterotypic organoids of pancreatic ductile adenocarcinoma, that can further trigger the release of encapsulated chemotherapeutics following NIR photodynamic activation [51]. This chemotherapy (oxaliplatin, gemcitabine hydrochloride, and 5-fluorouracil) loaded liposomal formulation significantly increased the antitumor efficacy of the combination therapy, with respect to the chemotherapy-free liposomes [51].

In this study, a NIR-triggered theranostic approach is developed for combined cancer treatment under complementary fluorescence and optoacoustic monitoring. We use photoactivatable multi-inhibitor liposomes (PMILs), carrying a lipid-anchored derivative of the photosensitizer benzoporphyrin (BPD) in the bilayer and topoisomerase I inhibitor (Irinotecan: IRI) entrapped through a transmembrane gradient of an entrapping agent in the inner core of nanoliposomes. It was hypothesized that the developed PMILs can uniquely co-deliver theranostics to tumors and, thus, provide a cooperative enhancement of photodynamic and chemotherapeutic regimens. To confirm the hypothesis, murine CT26 tumors in a pre-clinical mouse model are treated with PMILs. Complementary intra- and post-procedure OA and FL monitoring are performed non-invasively in vivo to track the immediate (hours-scale) effects of the therapy on tumor vasculature and correlate them with histological data. Tumor growth inhibition is assessed as a standard metric of tumor response.

## 2. Materials and Methods

### 2.1. Materials

All lipids 1-arachidoyl-2-hydroxy-sn-glycero-3-phosphocholine (20:0 Lyso PC), 1,2-distearoyl-sn-glycero-3-phosphocholine (18:0 PC (DSPC)), 1,2-dioleoyl-sn-glycero3-phospho-(1’-rac-glycerol) (sodium salt) (DOPG), cholesterol, 1,2-distearoyl-sn-glycero-3-phosphoethanolamine-N-[methoxy(polyethyleneglycol)]-2000 (DSPE-mPEG-2000), were obtained from Avanti Polar Lipids, Inc. (Alabaster, AL, USA). Verteporfin (Benzo porphyrin; BPD) was purchased from US Pharmacopeia (Rockville, MD, USA). Irinotecan hydrochloride salt, trihydrate was obtained from LC Laboratories.

### 2.2. Preparation of Photoactivatable Liposomes (PALs)

Prior to liposomal preparation, the photosensitizer Benzoporphyrin derivative (BPD) was anchored to the 1-arachidoyl-2-hydroxy-sn-glycerol-3-phosphocholine (20:0 Lyso PC) through Steglich esterification [51,67,68,69,70]. The purified product 20:0 Lyso PC-BPD (lipidated-BPD) was stored at −20 °C in the dark. Photoactivatable multi-inhibitor liposomes (PMILs) were prepared by the thin-film hydration and extrusion method [50,59,60,61,62]. All liposomes have a similar lipid composition. The lipids 18:0 PC (DSPC) (790.14 g/mol), Cholesterol (386.65 g/mol), and DSPE-mPEG-2000 (2803.79 g/mol), were mixed at a ratio of 53.2:45.9:0.3 mol% with 200 nmoles of lipid-anchor (BPD-PC; 1249.72 g/mol). The mixture was dried under a gentle nitrogen gas flow to remove the chloroform and to form a thin film. Thin lipid films were kept under vacuum for 1 h to remove any residual chloroform. Dried lipid films were added with 50% (w/v) of ethanol and hydrated with 10 volumes of pre-heated solution (250 mM) of an ammonium salt of sulfate ((NH_4_)_2_SO_4_), in screw-capped glass scintillation vials at 65 °C (water bath). These multilamellar vesicles (MLVs) were subjected to 5 freeze-thaw cycles, consisting of incubation at 65 °C (5 min; water bath) and incubation in liquid nitrogen (3 min). MLVs were then sequentially extruded (15 times) through two polycarbonate membranes (100 nm pore size, Avanti^®^ Polar Lipids, Inc.) using a mini-extruder system (Avanti Polar Lipids, Inc.) to prepare small unilamellar vesicles (LUVs). Extra Liposomal (NH_4_)_2_SO_4_ was removed and the external aqueous phase of LUVs was exchanged on a Sepharose CL-4B size exclusion column packed with Sepharose CL-4B (Sigma-Aldrich, St. Louis, MO, USA) pre-equilibrated with HEPES-buffered dextrose (5 mmol/L HEPES, 5% dextrose, pH 6.5).

### 2.3. Irinotecan (IRI) Entrapment to Photoactivatable Liposomes

Photoactivatable multi-inhibitor liposomes (PMILs) were prepared by entrapping Irinotecan Hydrochloride Hydrate (IRI.HCl. 3H_2_O; 677.18 g/mol) into the photoactivatable liposomes (PALs). IRI.HCl. 3H_2_O solution (20 mg/mL) in HEPES-buffered dextrose (5 mmol/L HEPES, 5% dextrose, pH 6.5) was added to the purified PALs. The resulting mixture was further incubated at 65 °C (water bath) for 30 min in the dark and cooled on ice for 15 min to quench the reaction.

DSPE-mPEG2000 (to provide 4.2% surface coverage with PEG) and DOPG (for an anionic charge) micelles were formed by replacing the chloroform with HEPES-buffered saline (5 mmol/L HEPES, 145 mmol/L NaCl, pH 6.5) and were slowly added to the dilute suspension of the pre-formed liposomes (IRI entrapped PALs) at temperatures close to the transition temperature of the constituent lipids (65 °C; water bath) for 60 min in the dark to allow post insertion of the DSPE-mPEG2000 and DOPG micelles into PMILs. Unentrapped IRI was subsequently removed using dialysis (Spectra/Por Float-A-Lyzer G2 Dialysis; MWCO 100 kDa) overnight at 4 °C, against 1 L of HEPES-buffered saline (5 mmol/L HEPES, 5% dextrose, pH 6.5). Prior to dialysis, Float-A-Lyzer was activated in 10% ethanol and washed with Millipore water. Liposomes entrapped with IRI only (L-[IRI]) or BPD-PC only (PALs) were also prepared and purified similarly.

### 2.4. Physical Characterization

The molar concentration of BPD-PC (ε687 nm = 34,895 M^−1^ cm^−1^) and IRI (ε384 nm = 21,835 M^−1^ cm^−1^) was determined by diluting liposomes in DMSO and measuring the absorption spectrum using UV–visible absorption spectrophotometry. Entrapment efficiency was determined in all preparations by quantitating IRI and comparing the resulting IRI/phospholipid final ratio to its initial ratio. Hydrodynamic diameter (nm), polydispersity index (PDI), and ς-potential (mV) of all liposomal nanoconstructs were measured through a Zetasizer Nano ZS Dynamic Light Scattering Instrument (Malvern Instruments). Measurements were performed in triplicates and values were reported as mean and standard deviation.

### 2.5. Irinotecan (IRI) Release from PMILs

IRI release kinetics under dark conditions and photoinduced drug release for a specified time was measured using dialysis membranes in phosphate-buffered saline (PBS). Ten percent fetal bovine serum (FBS) was added to each dialysis tube (Spectra/Por; 100 kDa cutoff) and was kept under dark conditions in an incubator shaker at 37 °C. A 690 nm diode laser (High Power Devices, Inc.) was used for all NIR irradiation experiments. During dialysis, samples were collected periodically. IRI was quantified through spectrophotometry (ε384 nm = 21,835 M^−1^ cm^−1^).

### 2.6. Experimental Setup for FL and OA Imaging

**Figure 1:**
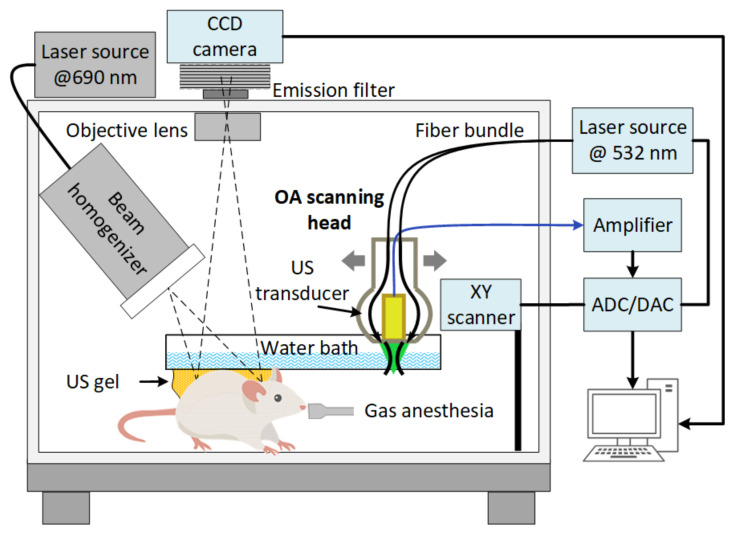
The scheme of the imaging setup for complementary FL and OA monitoring of PDT.

**Figure A1:**
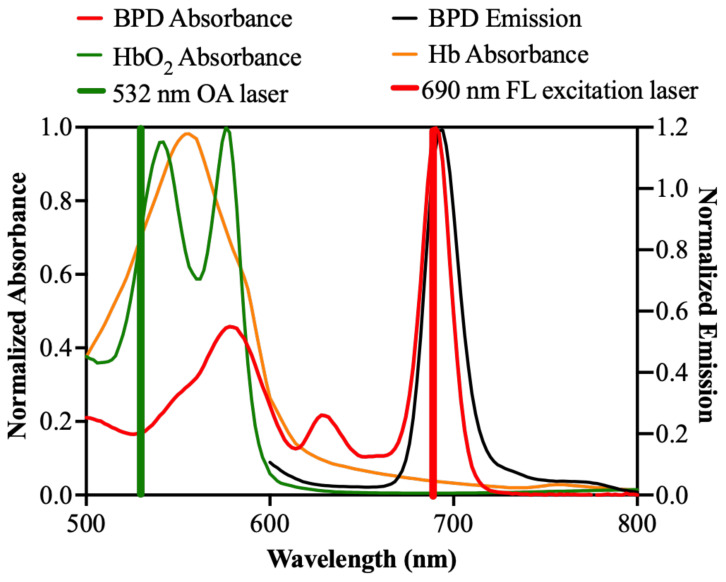
Absorption spectra of oxy- and deoxyhemoglobin in comparison with absorption and emission spectra of BPD. The laser lines for OA imaging (532 nm) and FL excitation (690 nm) are also added to the graph.

The experimental setup (Figure 1) for bimodal optical imaging was based on our previous version of the OA and FL imaging setup described in general in [71,72]. OA imaging employed a concept of dark-field acoustic-resolution photoacoustic microscopy (AR-PAM), where an OA head capable of scanning in two dimensions was represented by a conical fiber-optic illumination system [73] combined with a spherically focused acoustic detector made of a PVDF film [74]. The wideband ultrasound PVDF antenna with 5–40 MHz bandwidth provided the ability to image tumor vasculature [75] at up to 2 mm depth with axial/transverse resolution of 38/50 μm. For OA imaging, a 532 nm laser with a 2 kHz repetition rate and 0.2 mJ pulse energy was used. The wavelength is chosen according to high hemoglobin absorption (Figure A1). OA A-scans corresponding to discrete XY positions of the scanning head were recorded by a 16-bit analog-to-digital converter (ADC), a CSE1622 (Gage, Lockport, IL, USA). The same board was operated also as a digital-to-analog (DAC) converter to operate the XY scanner. The scanning step was 25 µm in the X and Y directions. The scanning area was 10 × 10 mm^2^. The acquisition time for every 3D data set was ~5 min. 

For FL imaging the laser diode sources at the wavelength of 690 nm corresponding to the absorption band of BPD (Figure A1), and an optical filter 725/40 nm for detection of the BPD fluorescence emission were used. A quasi-uniform distribution of light irradiation at the object surface was formed using an ED1-S20-MD diffuser (Thorlabs, Newton, NJ, USA). The cooled CCD camera ATIK314L+ (Artemis CCD Ltd., Norwich, UK) with motorized filter wheel (Finger Lakes Instrumentation, Lima, NY, USA) and Sigma lens (focal length: 28 mm; f/1.8) was employed for fluorescence detection.

### 2.7. Numerical Analysis of FL Images

For each mouse from the PALs and PMILs groups the series of fluorescence images were registered at reference times of the experiment: before injection (as a background signal), after drug accumulation in tumor (prior to PDT procedure), immediately after PDT procedure, and at 4, 24, and 72 h after PDT. Averaging fluorescence signals over tumor ROI and in surrounding non-irradiated tissue allowed tracking the PS accumulation, photobleaching, and the changes in fluorescence intensity in 24 h after PDT and comparing these results for tumor and non-irradiated tissue.

Accumulation of PS, A_PS_ Was Calculated as Follows

(1) $$A_{PS}=\left(I_{0}-I_{b}\right)/I_{b}$$

where *I_b_* is the background intensity (autofluorescence + stray light measured before PS injection) averaged over ROI (tumor or surrounding non-irradiated tissue); *I*_0_ is the fluorescence intensity averaged over the same ROI prior to PDT. This value is proportional to the accumulated PS concentration in the ROI.

Photobleaching of PS in the Tumor, B_PS_ Was Calculated as Follows

(2) $$\;B_{PS}=\left(I_{0}-I_{1}\right)/\left(I_{0}-I_{b}\right)$$

where *I*_1_ is the fluorescence intensity averaged over ROI after PDT. This value characterizes the efficiency of light absorption by PS accumulated in the irradiated region. The case of *B_PS_* = 0 indicates no bleaching occurred, while *B_PS_* = 1 indicates complete PS bleaching. A negative value of *B_PS_* can be associated with the activation of blood circulation and enhanced PS delivery as a result of the procedure manifested by an increase in FL signal. 

The relative change in the fluorescence intensity typically observed in the tumor 24 h after PDT, *C*_24_, is calculated as

(3) $$\;C_{24}=\left(I_{24}-I_{0}\right)/\left(I_{0}-I_{b}\right)$$

where *I*_24_ is the fluorescence intensity averaged over ROI measured 24 h after PDT. Calculations of the *B_PS_* and *C*_24_ values were performed for ROIs encompassing the tumor region and the surrounding non-irradiated tissue that was used as a reference.

### 2.8. Numerical Analysis of OA Images

A recently developed three-dimensional OA image processing algorithm [76] was applied to reconstructed and Frangi filtered OA data. After the acquisition, a two-dimensional delay-and-sum reconstruction [77] in the frequency domain [78] was applied to raw OA data consequently to B-scans in XZ direction and YZ direction. Frangi 3D filtering from the k-Wave toolbox [79] was applied to the reconstructed OA data. Frangi 3D filtering enabled the separation of inextricable vascular (cylindrical) structures in a diameter range of 25, 50, 100, and 150 µm, against the background of ‘non-vascular (flat and spherical) structures. The OA reconstruction and Frangi-filtration algorithms were applied in the same way as previously reported [76,80]. The proposed algorithm was employed to calculate the total amount of independent tree structures per volume unit (vesselness index VI) in a tumor tissue based on a graph defined by the skeletonized image. The tumor area was selected manually based on reconstructed OA image morphological data. A binarization procedure was applied to the OA image only in the tumor area defined by the mask. The following skeletonization, which removed the information about vessel diameter, and graph construction allowed defining the VI as a number of unique vascular trees normalized by the tumor volume associated with average vessel number per mm^3^.

### 2.9. In Vivo PMILs Penetration and NIR Photodynamic Activation

All experiments on animals were performed in accordance with relevant guidelines and regulations and documented following the ARRIVE guidelines.

Female BALB/c mice (8 - week-old, 18–20 g body weight) were obtained from the Pushchino nursery for laboratory animals (Moscow, Russia). Murine colorectal tumors CT26 were generated by subcutaneous implantation of 2 × 10^5^ cancer cells in 100 μl of phosphate-buffered saline (PBS) into the left thigh. 

The mice bearing CT26 tumors were randomly divided into four experimental groups: (1) untreated; (2) L-[IRI]; (3) PALs, and (4) PMIL, 3–4 animals per group in long-term growth experiment, and 3 animals per group in imaging/histology experiment. When the tumor volume reached ~25 mm^3^, the mice were intravenously injected with L-[IRI], or PALs, or PMILs at a dose of 10 mg/kg of IRI equivalent, and 0.25 mg/kg of BPD equivalent. fifteen minutes following the administration of PALs or PMILs, tumors were irradiated with a homogeneous optical beam at 690 nm corresponding to the absorption maximum of BPD using the same diode laser as used for FL imaging, but with the increased power (0.75 W output power from the optical fiber). The power density on the tissue surface was 100 mW/cm^2^, and the light dose was 100 J/cm^2^. Laser irradiation of mice was performed outside the dark imaging chamber with the murine skin surface cleaned and dried to prevent the possible increase in optical fluence in the tissue occurring by the refractive index matching of the tissue surface with the ultrasound gel applied on it [81].

### 2.10. Monitoring of Therapeutic Effect on the Vascular and Cellular Components of Tumor Tissue

**Figure 2:**
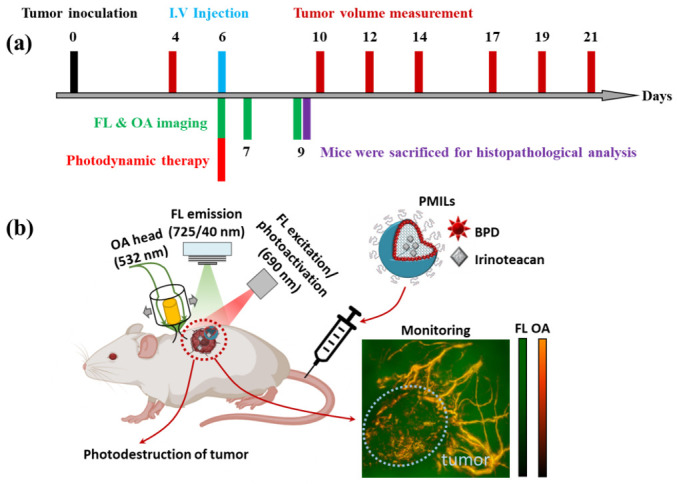
(**a**) Sequence of CT26 tumor implantation, FL and OA imaging, treatment with PAL, L-[IRI], or PMIL, (**b**) Schematic representation of the mechanism of action of photoactivatable multi-inhibitor liposomes (PMILs) following photoactivation. The photosensitizer tumoral uptake can be monitored with FL imaging, while the photodestruction of the tumor blood vessels can be monitored with OA imaging.

Tumor size was measured with a caliper in three mutually perpendicular directions every 2–4 days starting from the 4th day of tumor growth until day 21 (Figure 2a). The tumor volumes were calculated by the formula V = (4/3) × π × (a/2) × (b/2) × (c/2), where a, b, and c are the three perpendicular tumor diameters. The tumor growth inhibition index was defined as (1 − (mean volume of treated tumors)/(mean volume of control tumors)) × 100%) [82].

The photosensitizer tumoral uptake following PMILs photoactivation can be monitored with FL imaging, while the photodestruction of the tumor blood vessels can be monitored with OA imaging (Figure 2b). Prior to the optical monitoring experiment, the skin of the tumor zone was shaved to avoid image artifacts caused by hair. Animals were anesthetized with 1.5% isoflurane in 100% oxygen with a flow of 0.1 L/min and fixed in the customized supporting plate. In the PMILs and PALs groups, OA imaging was performed prior, 0.5, 4, 24, and 72 h post-PDT. FL imaging was carried out before, 10, and 15 min after the injection of the photosensitizer and after PDT at different time points (5 min, 30 min, 4, 24, and 72 h). In the L-[IRI] group and the untreated group, OA imaging was performed at 0, 24, and 72 h.

### 2.11. Morphological Study

Histological analysis and immunohistochemistry (IHC) of tumor tissues were performed on the 9th day after tumor cells inoculation. The excised tumors were embedded in paraffin and 7 μm thickness slides were prepared from each tumor. For histological analysis specimens were stained with hematoxylin and eosin (H&E). The histopathology examination included both the general structure of the tumor tissue and the character of the vasculature. The percentage of the hemorrhage area relative to the slice area was calculated using the ImageJ software (NIH, Bethesda, MD, USA). For IHC analysis, tissue slices were stained with monoclonal rabbit antibodies CD31 (ab225883, Abcam, Cambridge, UK), following the manufacturer’s protocol, and then incubated with secondary antibodies conjugated with horseradish peroxidase (ab6728, Abcam, UK). Tissue slices were examined using a Leica DM2500 microscope.

### 2.12. Statistical Analysis

The mean values (M) and standard errors of the mean (SEM) were calculated for tumor volume; the average values and standard deviations (SD) were calculated for fluorescence parameters (PS accumulation, photobleaching, and signal change 24 h post-PDT) and percentage of hemorrhages. For statistical analysis, the IBM SPSS Statistics software was used. The Bonferroni post hoc test was employed to compare different treatment groups, with *p* < 0.05 being considered statistically significant. The Pearson correlation coefficient was calculated to assess the correlation between the vesselness index (VI) and the percentage of hemorrhages 72 h after treatment.

## 3. Results

### 3.1. Design, Preparation, and Characterization of Photoactivatable Multi-Inhibitor Liposomes (PMILs) for Imaging-Based Treatment Monitoring

**Figure A2:**
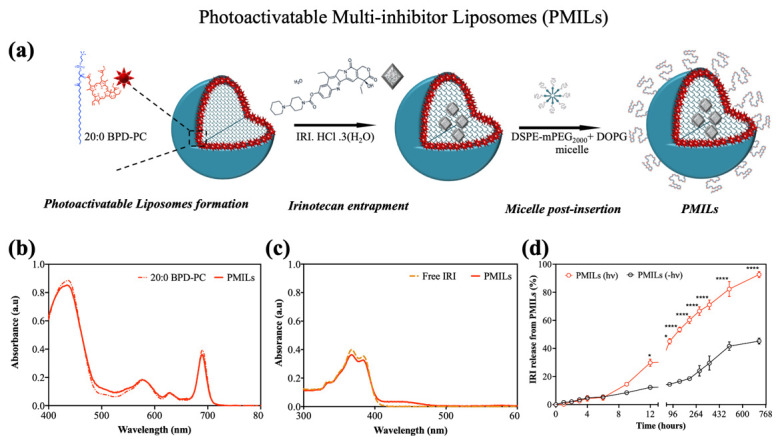
(**a**) Schematic representation of photoactivatable multi-inhibitor liposomes (PMILs) preparation chemical structures of the lipidated variant of BPD conjugated to 20:0 PC (20:0 PC-BPD) and IRI.HCl. (**b**) UV-Visible absorption spectra of 20:0 BPD-PC (**c**) free IRI.HCl and PMILs. (**d**) Photo-triggered IRI release from PMILs versus IRI release under dark conditions. PMILs were triggered with NIR light (40 J/cm^2^, 150 mW/cm^2^) at 37 °C. Data presented as mean ± s.e.m. (*n* = 3 technical replicates each performed with independent PMILs preparation. (One-way ANOVA with Tukey’s multiple comparison test, **** = *p* < 0.0001, * = *p* < 0.05).

Photoactivatable multi-inhibitor liposomes (PMILs) were prepared with the lipid-anchored photosensitizer BPD formulated within the liposomal bilayer (Figure A2a). The resulting PMILs exhibited characteristic absorbance peaks at 384 and 687 nm corresponding to IRI and BPD-PC, respectively, suggesting that lipid-anchoring of BPD did not affect the absorption properties of BPD (Figure A2b). Moreover, IRI also did not show any spectral shift after entrapment (Figure A2c). The PMILs exhibited an average hydrodynamic size of 131.6 ± 2.1 nm and polydispersity index (PDI) of 0.05 ± 0.01 demonstrating that a narrow size distribution and monodispersity were well maintained in all nanoconstructs. **ζ** -potential of PMILs was recorded between −14.9 ± 1.04 to −16.9 ± 0.92 mV, which was suggestive of an anionic charge in all PMILs prepared (Table 1). **ζ**-potential prevents the aggregation of nanoliposomes and thus charged liposomes offer great stability on storage. A consistent **ζ**-potential is also essentially required to minimize variability in cellular uptake of different nanoformulations by the tumor cells [51]. The stability of PAL, L-[IRI], and PMIL was assessed periodically by measuring the size and PDI during storage at 4 °C. No significant change was observed even after storage at 4 °C over a period of 30 days. These findings are consistent with our previous work, demonstrating no significant size change in PMIL over 2.5 months of storage at 4 °C [51,53].

IRI release from PMILs was measured periodically at 37 °C. Photodynamic activation resulted in 2.4-fold higher IRI leakage from PMILs at 12 h post-incubation in comparison to the dark conditions (Figure A2d). This suggests that irradiation disrupts the integrity of the PMIL, thereby releasing the drug. Enhanced leakage stability under dark conditions was observed, with only 45% of the drug release at day 30 as compared to phototrigered release (93%) (Figure A2d). This PMIL design can facilitate encapsulation of multiple drugs, protecting enclosed cargo from hydrolysis and break down, thus, offering new prospects for cancer theranostics [51,53]. When imparting photo-induced cytotoxicity along with the photoinitiated release of the IRI, a single PMIL treatment offers spatiotemporally synchronized combination therapy to suppress tumor regrowth.

### 3.2. Combination Therapy Causes Tumor Growth Inhibition

**Figure 3:**
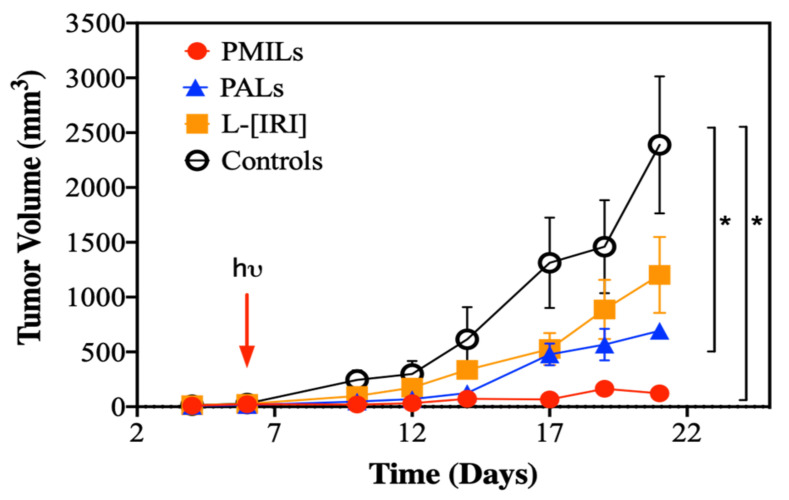
CT26 tumor volume dynamics after PDT with PALs, chemotherapy with L-[IRI], and combined therapy with PMILs (M ± SEM). Untreated animals served as control. Arrow indicates the day of treatment. * Indicates statistically significant differences between the groups (*p* < 0.05, one-way ANOVA with Bonferroni correction).

Figure 3 shows the CT26 tumor growth dynamics between days 4 to 21 after tumor inoculation. During the investigation period, the volume of untreated tumors increased from 14.4 ± 1.9 mm^3^ to 2390 ± 880 mm^3^. At the end of the monitoring period on the 21^st^-day post tumor inoculation, the tumor volumes were 1200 ± 600 mm^3^ for the L-[IRI] group, 460 ± 330 mm^3^ for the PALs group, and 60 ± 60 mm^3^ for the PMILs group. A statistically significant difference between treated and untreated animals on the 21st day was revealed for PALs (*p* = 0.03) and PMILs groups (*p* < 0.01). The PMILs group demonstrated a higher tumor inhibition effect in comparison to L-[IRI] and PALs. On day 21, the tumor growth inhibition index was 97% for the PMILs group, 81% for the PALs group, and only 50% for the L-[IRI] group.

### 3.3. In Vivo FL Imaging Shows Accumulation, Photobleaching, and Re-Accumulation of BPD-Containing Liposomes

**Figure 4:**
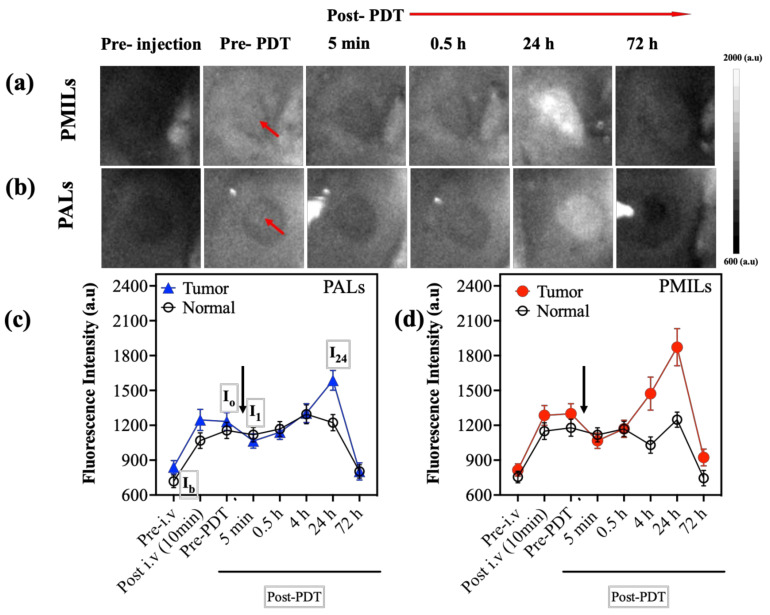
FL monitoring of PDT. Representative fluorescence images were obtained at different time points for mice from PMILs (**a**) and PALs (**b**) groups. Red arrows show the tumor nodes. Dynamics of fluorescence signals in tumor (blue and red curves) and surrounding normal tissues (black curves) calculated for a mouse from PALs (**c**) group and PMILs group (**d**). Black arrows correspond to the start of light exposure. Mean ± SD.

Using FL imaging, in vivo accumulation of PALs and PMILs was observed in all mouse tumors and the surrounding tissue (Figure 4). PDT resulted in photobleaching of the PS in the irradiated area as expected, and consequently, in the decrease in the fluorescence intensity immediately after PDT. The photobleaching effect (corresponding to B_PS_ > 0) was observed in all irradiated tumors except for one from the PMILs group. After 4 h, the fluorescence intensity in the tumors recovered to the pre-PDT value and continued to increase until 24 h in both groups. In the PMILs group, the increase in the FL intensity was more pronounced. In non-irradiated surrounding tissues, the FL signal on the next day after treatment decreased, which is likely associated with the PS elimination from the body. Complete elimination of both PALs and PMILs and a decrease in the signal to a pre-injection level were detected at 72 h. Quantitative assessments of the FL signal in the tumors and surrounding tissue are summarized in Table 2.

The increase in fluorescence signal 24 h post-PDT can be attributed to PS release from the liposomal bilayer. To study this assumption, an additional model experiment on the PALs dissolved in DMSO + intralipid solution was conducted. To study this, the experiment of the PALs dissolved in DMSO + Intralipid solution was conducted. A ~1.7-fold increase in the fluorescence signal was found after BPD release from the liposome shell due to the disappearance of the fluorescence quenching effect within liposomes (see Appendix A for details).

### 3.4. In Vivo OA Imaging Reveals the Growth of Vesselness Index in Response to Therapy with PMILs

**Figure 5:**
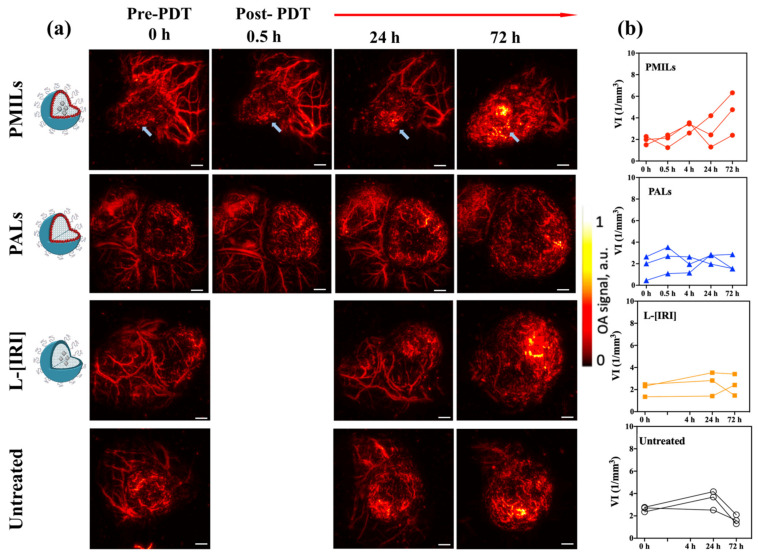
In vivo OA imaging of the tumors in different treatment groups (**a**) Representative angiographic OA images obtained for different groups of mice prior to treatment and in the follow-up. Blue arrows indicate the areas of formation of hemorrhages in the PMILs group. (**b**) The dynamics of the vesselness index (VI, 1/mm^3^) value in tumor calculated from OA images for each mouse in the groups aiming at the characterization of the observed changes in the microvasculature net as a result of treatment. Scale bar = 1 mm.

OA imaging of the tumors was performed to monitor the dynamic changes in the microvascular structure due to therapies. OA imaging was able to distinguish between tumor vasculatures and surrounding tissues (Figure 5a). Tumor tissue is characterized by smaller and more tortuous vessels forming a denser vessel net as compared to the vessel structure in the peritumoral area.

After treatment with PMILs, a strong increase in the OA signal was observed in the tumor (Figure 5 first row). An increase in the vesselness index VI (Figure 5b, PMILs group) was detected from 24 h to 72 h. The rise in VI can be explained both by an increase in the number of blood vessels and by the formation of hemorrhages. Note, that following the applied algorithm of VI calculation, hemorrhages and destructed vessel fragments are considered as independent vessel trees, hence, as a result, the VI value increases if both features are present. Presumably, the increase in VI in the PMILs treated group can be connected to the efficient destruction of vessels upon Irinotecan release from the liposomal core. In contrast, the untreated group demonstrated a decrease in this parameter in the same period, indicating the preservation of the intact vessel net structure in the tumors and lower vesselness. For the L-[IRI] and PALs groups, no significant alterations in the VI value for 72 h were observed, which, presumably, is the result of two competing effects: tumor development and response to treatment procedure, since no full response was observed in these groups according to histological data.

### 3.5. The Results of Optoacoustic Imaging Correlate with Histology

**Figure 6:**
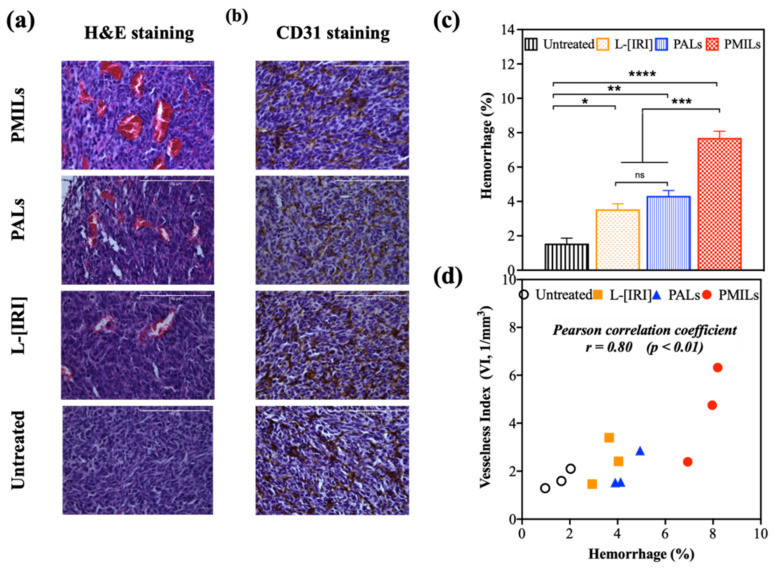
Histopathological analysis of CT26 tumors in the 72 h after treatment with PMILs group, PALs, or L-[IRI]. (**a**) H&E staining; the scale bar is 150 µm. (**b**) IHC for CD31; the scale bar is 150 µm. (**c**) Percentage of hemorrhage in H&E-stained slides (mean ± SD); * indicates statistically significant differences between the treatment groups (**** = *p* < 0.0001, *** = *p* < 0.001, ** = *p* < 0.01, * = *p* < 0.05, one-way ANOVA with Bonferroni correction). (**d**) Vesselness index VI (according to OA) versus percentage of hemorrhages (according to H&E staining). Marks show the measurements for each mouse.

Standard histopathological analysis and IHC for endothelial marker CD31 were performed 72 h after treatment (Figure 6). In the untreated group, the tumor tissue had a dense structure and consisted of polymorphic cells of different sizes with large round or oval nuclei containing diffusively distributed chromatin and 1–2 nucleoli. The slightly basophilic cytoplasm formed a thin ring around the nucleus. There was a large amount of mitosis, there were no foci of necrosis. A small number (up to 10%) of cells with dystrophic changes, such as vacuolization of the cytoplasm and condensation of chromatin, was presented. The stroma was poorly manifested. The hemorrhage area was 1.6 ± 0.5%.

In the L-[IRI] group the density of the tumor structure slightly decreased with respect to the untreated group. Hyperemia was more pronounced; hemorrhages were noted in the center of the tumor. Hyperchromic nuclei were more common, and separate small foci of necrosis were being formed. The hemorrhage area was 3.5 ± 0.6%.

In the PALs group, the density of the tumor structure slightly decreased as compared to untreated tumors. The number of mitotic cells decreased and the number of dystrophic cells with chromatin condensation increased. Vascular abnormalities, such as hemorrhages, disruption of individual vessels, and enlarged sinusoids, were observed. There were foci of necrosis in the center of the tumor. The hemorrhage area was 4.3 ± 0.5%.

In the PMILs group, the tumor tissue lost its dense structure in the center. Vascular abnormalities were more pronounced in comparison with other groups, including the surface area. Vascular thrombosis and hemolysis were observed. Pronounced hyperemia, destruction of the vessels, and hemorrhages were present. There were foci of necrosis and dystrophic cells with chromatin condensation. The hemorrhage area was 7.7 ± 0.8%. Large areas of hemorrhage 72 h after treatment were observed in all groups, however, the effect was most pronounced in the PMILs group.

Statistically significant differences in the percentage of hemorrhages in the tumor were observed between groups of untreated and treated animals (*p* < 0.01 for PALs group, *p* = 0.02 for L-[IRI] group, *p* < 0.01 for PMILs group) and between different groups of treatment (*p* < 0.01 between PMILs and PALs, as well as between PMILS and L-[IRI]). Quantification of H&E-stained images was in good agreement with OA data, i.e., a strong positive correlation (*r* = 0.80, *p* < 0.01) was revealed between the values of hemorrhage area and vesselness index (Figure 6d). The results of IHC analysis demonstrated a comparable amount of endothelial marker CD31 in all groups suggesting that the effect observed by OA primarily originates from the appearance of numerous hemorrhages formed post-treatment and is not governed by the newly developed vessels.

## 4. Discussion

The recognition of multiple pathways supporting tumor growth has led to an acceptance of combination treatments to control the disease. In this context, it has been shown previously in several studies [51,53,65,83] that the simultaneous inhibition of these mechanisms is more effective in both primary tumor control and metastatic spread. When the therapeutic agents are administered in nanoconstructs to target multiple pathways, the agents need to be delivered at the same place at the same time to increase the chance of synergy and thereby, disease control [53]. In addition to a combination of therapeutics, simultaneous monitoring of changes in tumor biology to predict treatment outcomes especially at early stages is very valuable [24,84]. Improvements in the design of multi-modal nanoformulations have facilitated the specific delivery of theranostics in vitro and in vivo, while improvement in optical imaging techniques has enabled monitoring of drug uptake and dynamic changes in tumor vasculature.

The encapsulation efficiency of the chemotherapeutics within the PMILs also impacts the treatment outcome. The use of rationally designed liposomal-based drug delivery systems becomes an alternative strategy to reduce toxicity and improve the efficacy of treatment. In this study, IRI.HCl. 3H_2_O is actively loaded using a transmembrane gradient of (NH_4_)_2_SO_4_, as this approach provides stability of drug loading without requiring specific pH to be maintained during the preparation of liposomes [85]. Liposomes can be tailored to entrap higher payloads (e.g., antibiotics, small molecules, fluorescent dyes). Transformative approaches have also been explored to custom design liposomal carriers for IRI delivery in robust preclinical models. More recently, silicasome (lipid bilayer mesoporous silica nanoparticle) has shown an improved IRI loading capacity and stability over the liposomal carrier model. Silicasome demonstrated improved pharmacokinetics, substantially improved efficacy, increased survival, and reduced bone marrow and GI toxicity compared to the free drug and Onivyde and tumor drug content over free drug and Onivyde, in orthotopic colon cancer model [86,87]. Employing an alternate approach, it has been shown by Chen et al. [88] that embedding silica-coated nanobowls in the hydrophilic cavity, thus by providing mechanical support, can enhance drug leakage stability in blood circulation. 

In this study, a combination of optically triggered PMILs with OA imaging and FL imaging geared towards image-guided cancer theranostics was shown. Specifically, the effects of BPD-based PDT in combination with IRI, using new liposomes-based nanoconstructs PMILs are investigated on a preclinical mouse-based tumor model. A comparative analysis of multi-inhibitor therapy with the relevant single-agent treatments revealed that PMILs demonstrate the highest tumor inhibition effect in comparison with PDT alone (PALs) or chemotherapy (L-[IRI]) alone. PALs have a better therapeutic effect as compared to L-[IRI]. These results are in complete agreement with the previously published reports [89] on pancreatic cancer models. Increased tumor growth inhibition was also revealed for treatment with liposomes containing BPD and IRI using a light dose of 75 J/cm^2^ (100 mW/cm^2^) and the synergetic effect was explained by PDT-induced decrease in the efflux of IRI from the cell, suppression of PDT-induced activation of monocarboxylate transporter 4 expressions and increased apoptosis [62]. It should be noted that to achieve complete inhibition of tumor growth using IRI, much higher doses are required, applied in several courses to avoid side effects [90]. In combination with PDT via PMILs, high treatment efficiency can be achieved that reduces the toxic exposure to the organism significantly.

In order to compare the immediate response of different therapeutic strategies in vivo, a system for complementary FL and OA imaging at the whole-tumor scale was developed. While FL imaging is an easy-to-implement technique widely used for PDT monitoring in preclinical and clinical studies to analyze drug accumulation and its preservation in the blood flow based on its fluorescence properties. OA imaging is a complicated but rapidly developing technology that has allowed for vascular response analysis utilizing hemoglobin high optical absorption.

In this study, FL imaging showed the accumulation of the PS and photobleaching after tumor laser irradiation for all mice in the PALs and PMILs group. It is worth mentioning that a strong (2–3 times) increase in the fluorescence signal in the tumor was observed 24 h after the procedure in contrast to the naturally decreasing fluorescence signal in the non-irradiated area. Presumably, this phenomenon can be explained by the two complementary factors: (1) the effects of PS release from the liposome shell resulting in the fluorescence increase due to disappearance of fluorescence quenching effect in the PALs and PMILs liposomal forms; (2) increased tumor vascular permeability and PS extravasation in tumor tissues as a result of PDT. The first effect was observed in a separate in vitro study described in the Appendix A, which revealed a 1.7-fold increase in fluorescence intensity upon BPD release from a liposome shell for PMILs in PBS solution with a scattering medium. The second effect was previously observed in pilot animal studies where PDT improved drug delivery to the tumor [82] that resulted in better tumor response to therapy. This effect is likely to occur in this study because the C_24_ value (Table 2) was several times higher in the tumor area in comparison to surrounding tissues.

OA imaging enabled us to monitor the vascular network structure of the tumors. OA images demonstrated differences in the vessel structure of the tumors compared to normal tissue. Tumor tissue is characterized by smaller and tortuous vessels forming a denser vessel net as compared to the vessel structure in the surrounding tissues. To quantify the differences between groups in OA images, the vesselness index (VI), associated with vessel density, has been calculated. Our custom-written algorithm to quantify the OA images revealed a strong increase in the vesselness index in the PMILs group from 24 h to 72 h after the therapeutic procedure. The observed effect is assumed to originate from hemorrhages formed post efficient destruction of vessels upon Irinotecan release from the liposome. The increase in OA signal due to hemorrhage was evaluated as additional vessel structures by the developed algorithm. In contrast to this, the untreated group demonstrated a decrease in the vesselness index value for the same time interval, which can be associated with differences in growth rate between vessels and tumor mass. The vascular system of CT26 develops more slowly than its cellular component; therefore, in some areas of the tumor node, extensive avascular zones are formed. Thus, at certain stages of neoplasm growth, the overall density of vessels may decrease. Uneven changes in vascular density during CT26 growth have also been noted by us and others [27,91]. No significant changes in the vesselness index value in L-[IRI] and PALs groups were detected, which can be explained by competing effects of natural tumor development and therapy impact. The observation of the largest increase in vesselness index value in the PMILs group agreed with the histological data showing the strongest therapeutic effect manifested by the largest number of hemorrhages in the PMILs group as compared to monotherapy PALs and L-[IRI] groups.

Vascular reaction to BPD-PDT and irinotecan has been already investigated previously in several ex vivo and in vivo studies. It was shown that PDT causes a decrease in blood flow, vascular occlusion, and hemorrhage formation and that the reaction was more pronounced after a short drug-light interval [13,14,24,92]. Specifically, at shorter drug-light intervals the photosensitizer is primarily confined to the vascular compartment, enabling vascular occlusion upon PDT. On the other hand, irinotecan has been proven to have an antiangiogenic effect on tumors [62,93,94,95]. The synergistic effect of vascular occlusion after PDT and the antiangiogenic action of irinotecan could have led to a reduction in the tumor volume after the combined treatment. Our previous work also showed that PDT enhances irinotecan retention in the cells via damage of drug efflux pumps also leading to enhanced therapeutic efficacy [62]. In this work, the reaction of the vascular bed to the combined effect of these drugs in comparison with monotherapies was studied in vivo using OA imaging for the first time. It was revealed that combination therapy resulted in a significant increase in the VI value on the third day after treatment.

The theranostic platform presented here consisted of the imaging and nano-drug delivery aspects that both require further development for clinical translation. OA imaging and FL imaging separately are paving the way into the clinical arena, particularly for imaging tumors [96] and aiding in surgical resection [97,98], respectively. Integrated OA and FL systems to facilitate simultaneous multi-modal imaging are yet to be developed. The PMILs nanodrug delivery system proposed here is a non-targeted nano system. Targeted nanoagents with single or multiple targeting moieties can enhance specificity to the tumor and warrant further testing for clinical translation. Overall, in this study, the potential of the image-guided theranostics in subcutaneous tumor models was demonstrated. Our future studies will involve evaluating the therapy in orthotopic and genetically engineered mouse models.

## 5. Conclusions

In conclusion, the PMILs provided light-triggered bimodal anticancer treatment by simultaneously delivering photosensitizer for PDT and chemotherapy. A new approach of combined OA and FL monitoring for non-invasive in vivo real-time tracing of photosensitizer kinetics and functional vascular effects in the treated area was demonstrated employing the PMILs. The vesselness index based on skeletonization of the reconstructed vessel net, obtained by OA imaging, was employed to quantify the vascular response to the treatment. The largest increase in the vesselness index occurred in tumors with PMILs administration and was in good agreement with the histological data that showed that the strongest therapeutic effect manifested by the percentage of hemorrhages in the PMILs treated mice as compared to PALs or L-[IRI] treated mice. Here BPD and IRI were utilized as two agents encapsulated in PDT, however, the PMILs platform is adaptable for delivering other photosensitizers and chemotherapy agents. It can be concluded that the newly developed system allows for an immediate evaluation of the response to treatment, warranting further studies in different tumor mouse models. Overall, these results suggest that simultaneous bimodal anticancer therapy employing PMILs (combination of PDT with chemotherapy), and treatment monitoring is an efficient way to improve cancer theranostics outcomes.

## Figures and Tables

**Table 1:** Physical characterization of PMILs.

Nanoliposomes	Hydrodynamic Diameter (nm)	Polydispersity Index (PDI)	ζ-Potential (mV)	Irinotecan Encapsulation Efficiency (%)
PMILs	130.5 ± 0.0	0.039 ± 0.0	−16.9 ± 0.9	97.0 ± 3.7
PALs	134.1 ± 0.7	0.049 ± 0.0	−14.9 ± 1.0	NA
L-[IRI]	130.3 ± 0.2	0.065 ± 0.0	−16.9 ± 1.0	95.0 ± 1.5

**Table 2:** Fluorescence parameters in CT26 mouse tumor and surrounding non-irradiated tissue calculated from FL imaging data. Mean ± SD.

Treatment Group	Tissue Type	Accumulation of PS (*A*_PS_)	Photobleaching of PS (*B*_PS_)	Change in FL Signal 24 h Post-PDT (*C*_24_)
PMILs	Tumor	0.78 ± 0.45	0.14 ± 0.32	3.13 ± 1.40
	Surrounding (non-irradiated) tissue	0.8 ± 0.44	0.06 ± 0.09	0.51 ± 0.6
PALs	Tumor	1.00 ± 0.82	0.39 ± 0.14	1.8 ± 1.2
	Surrounding (non-irradiated) tissue	1.1 ± 0.99	0.15 ± 0.17	0.01 ± 0.22

## Data Availability

The data used in this research is available from the corresponding author upon reasonable request.

## Appendix A

### Appendix A.1. Differences in Fluorescence Brightness of Free BPD and L-Form

It should be noted that fluorescence brightness increases when BPD is released from the liposomal form. This effect is caused by the fluorescence quenching effect for PALs. To describe this effect quantitatively, we have conducted the following model experiment. Two microcentrifuge tubes were filled with different mixtures: the first one contained the mixture of PMILs and PBS, and the second one contained the mixture of PMILs and DMSO. DMSO dissolved liposome shells, and BPD was released and remained dissolved in a free form in DMSO in the second tube. Both mixtures contained the same amount of PMILs, however, the fluorescence intensity in the second tube was 1.8 times higher than in the first one. Fluorescence intensity was measured by the fluorescence imaging setup described in the Materials and Methods section. An additional experiment was conducted using the same microcentrifuge tubes with the addition of the same amount of Lipofundin to imitate scattering biological tissue. This resulted in a slight decrease in the abovementioned ratio down to 1.7. Thus, when BPD is released from the liposomal form in an animal body, we should expect an increase in the fluorescence intensity by 1.7 times.
